# Month-Long Unclotted Hemoperitoneum on Edoxaban Confirmed by Laparoscopy: A Case Report

**DOI:** 10.7759/cureus.94618

**Published:** 2025-10-15

**Authors:** Yoichi Miyaoka, Shingo Shimada, Yuichi Yoshida, Ryoji Yokoyama, Akinobu Taketomi

**Affiliations:** 1 General Surgery, Abashiri-Kosei General Hospital, Abashiri, JPN; 2 Surgery, Otaru General Hospital, Otaru, JPN; 3 Gastroenterological Surgery, Hokkaido University Graduate School of Medicine, Sapporo, JPN

**Keywords:** doac, edoxaban, hemoperitoneum, laparoscopy, ptgbd, unclotted blood

## Abstract

Direct oral anticoagulants (DOACs) can alter the morphology and time course of intra-abdominal bleeding, yet intraoperative confirmation of prolonged unclotted hemoperitoneum is rarely described. We report a man in his early 70s taking edoxaban for atrial fibrillation who was admitted with acute cholecystitis and underwent percutaneous transhepatic gallbladder drainage (PTGBD). After spontaneous tube dislodgement, he developed hemoperitoneum; computed tomography (CT) showed bloody ascites without organized clots. Edoxaban was withheld for one week and then restarted, and he remained hemodynamically stable without transfusion. Approximately one month later, an elective laparoscopic cholecystectomy revealed a large volume of dark, free-flowing liquid blood throughout the peritoneal cavity, with no fibrin clots or organized hematoma and no active bleeding; the fluid was aspirated, and the postoperative course was uneventful. The findings suggest that factor Xa inhibition, by suppressing thrombin generation and fibrin polymerization, may impede secondary coagulation of blood already leaked into the peritoneal space, allowing intraperitoneal blood to persist in a liquid state for weeks even after hemostasis and temporary drug withdrawal. This case provides laparoscopic evidence that edoxaban-associated hemoperitoneum can remain unclotted for about one month, and it highlights the need for surgeons planning interval procedures in anticoagulated patients to anticipate liquid collections, prepare for early/repeated suction-irrigation, and operate with the expectation that identification of dissection planes and transection lines may be difficult.

## Introduction

Direct oral anticoagulants (DOACs) such as edoxaban are widely prescribed for the prevention of stroke in atrial fibrillation and for the treatment of venous thromboembolism due to their predictable pharmacokinetics and no requirement for routine monitoring; compared with warfarin, they reduce intracranial hemorrhage while shifting bleeding patterns toward gastrointestinal and intra-abdominal events [1,2]. A comparative pharmacological overview has also emphasized differences in bleeding profiles among DOACs [3]. Percutaneous transhepatic gallbladder drainage (PTGBD) is an established option for high-risk acute cholecystitis under the Tokyo Guidelines framework, but procedure-related complications include bile leakage, tube dislodgement, and bleeding from the transhepatic tract or gallbladder bed [4,5]. A case-based review has reported hemoperitoneum after PTGBD, highlighting its potential severity despite its rarity [6]. Physiologically, blood extravasated into the peritoneal cavity tends to coagulate, organize, and ultimately resorb over days to weeks; by suppressing thrombin generation and fibrin polymerization, factor Xa inhibition may impede secondary coagulation of blood that has already leaked into the peritoneal space [3]. Contemporary guidance also outlines reversal strategies for DOAC-related bleeding when necessary [7]. Several case reports describe intra-abdominal bleeding under DOAC therapy and spontaneous hemoperitoneum [8], and peritoneal bleeding related to PTGBD, including an autopsy case and a fatal self-removal case, has also been documented [9,10], with additional reports under factor Xa inhibitors [11,12]. However, intraoperative confirmation of hemoperitoneum persisting in a liquid, non-organized state for weeks after the bleeding source has been controlled appears to be absent. Therefore, we present this case to provide intraoperative evidence that edoxaban-associated hemoperitoneum can persist in a liquid, non-organized state for several weeks, emphasizing its implications for surgical planning and intraoperative management in anticoagulated patients.

## Case presentation

**Figure 1:**
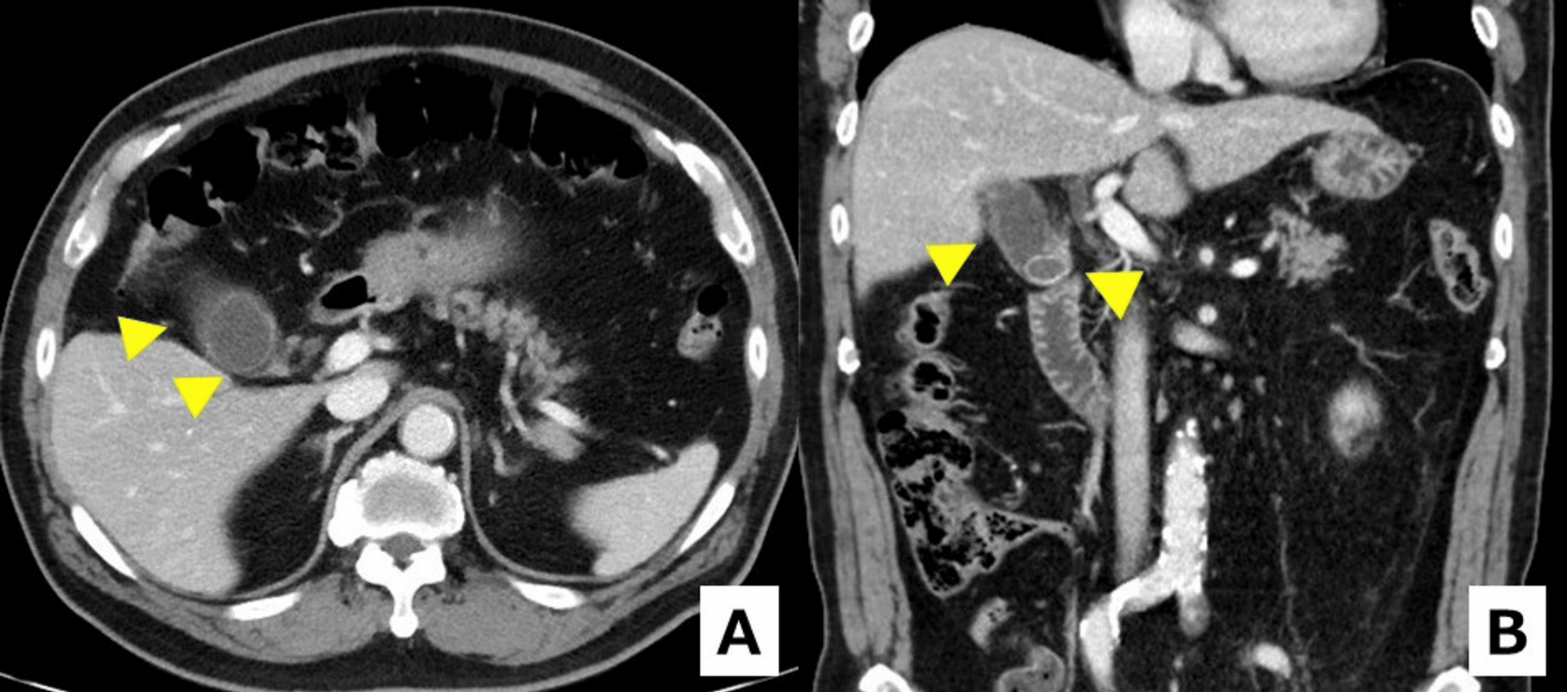
Admission contrast-enhanced CT demonstrating acute calculous cholecystitis (A) Axial and (B) coronal images show gallstones within the gallbladder, mural thickening, and pericholecystic inflammatory changes (arrowheads). CT: computed tomography

A man in his 70s with atrial fibrillation on edoxaban was admitted for acute cholecystitis. On admission, contrast-enhanced computed tomography (CT) demonstrated gallstones, gallbladder wall thickening, and pericholecystic inflammation consistent with acute calculous cholecystitis (Figure 1).

**Figure 2:**
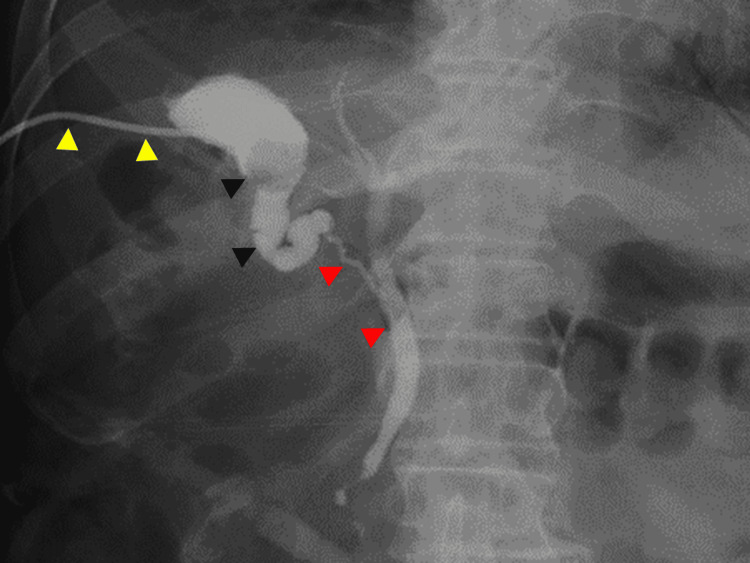
Cholangiography via the PTGBD tube Yellow arrowheads indicate the PTGBD tube. Black arrowheads outline the gallbladder and gallstones. Red arrowheads indicate the cystic duct and the common bile duct. No contrast extravasation is seen. PTGBD: percutaneous transhepatic gallbladder drainage

Because of surgical risk at presentation, percutaneous transhepatic gallbladder drainage (PTGBD) was performed. Cholangiography via the PTGBD tube delineated the gallbladder, cystic duct, and common bile duct without contrast extravasation (Figure 2).

**Figure 3:**
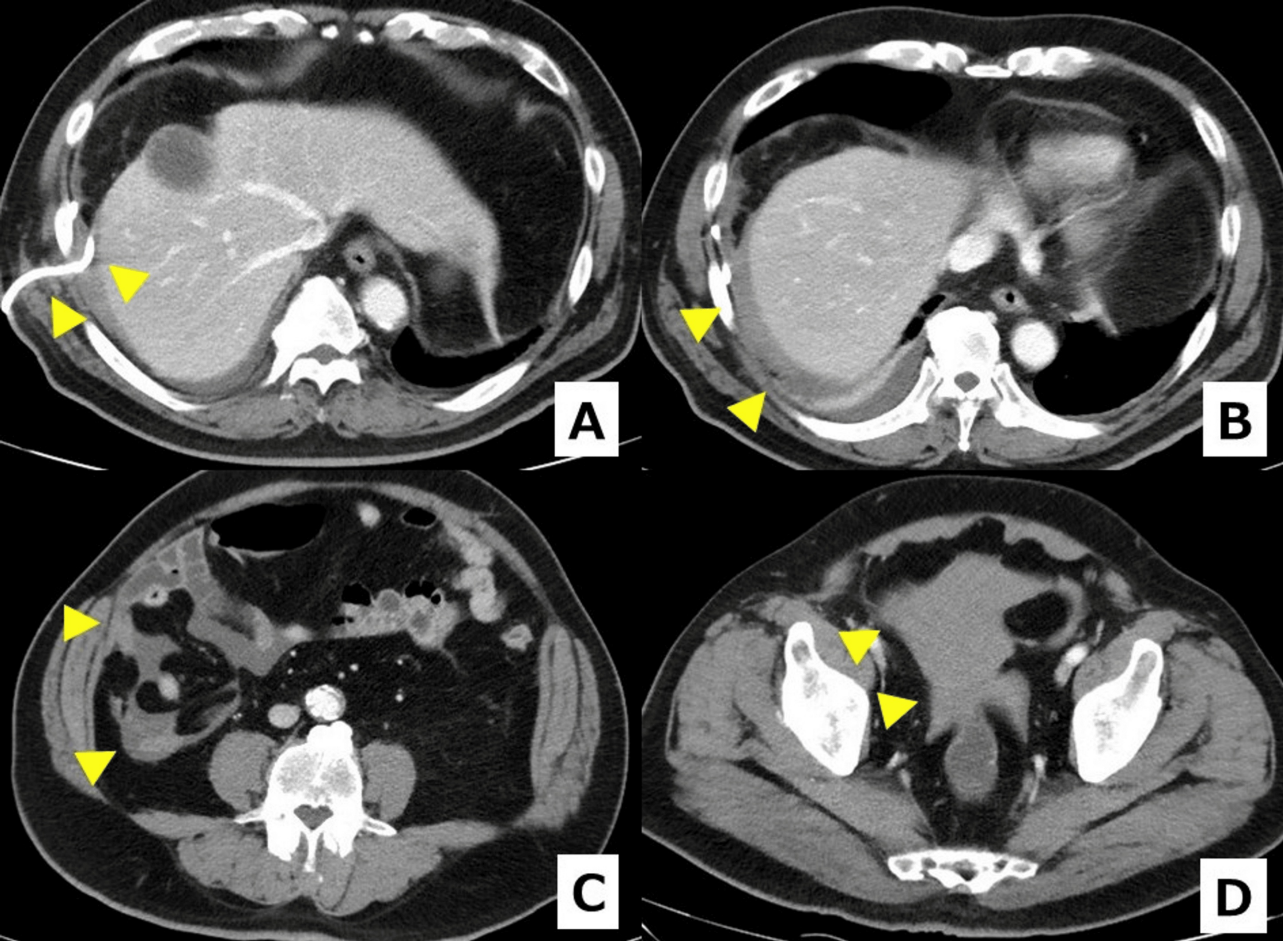
Contrast-enhanced CT after spontaneous PTGBD tube dislodgement (A) Arrowhead: PTGBD tube slipped out of the gallbladder. (B-D) Arrowheads: hemoperitoneum as liquid attenuation bloody ascites layering in the subhepatic space, right paracolic gutter, and pelvis. Attenuation is compatible with acute blood (~30-45 HU), supporting bloody ascites, with no organized hematoma. CT: computed tomography, PTGBD: percutaneous transhepatic gallbladder drainage, HU: Hounsfield units

Approximately five days after PTGBD, the drainage tube spontaneously dislodged. The patient remained hemodynamically stable. Contrast-enhanced CT obtained at that time demonstrated diffuse liquid hemoperitoneum layering in dependent spaces (subhepatic, right paracolic, and pelvic), with attenuation values compatible with acute blood (~30-45 Hounsfield units (HU)), without active extravasation or organized hematoma (Figure 3).

Laboratory tests showed a gradual hemoglobin decline from 16.1 g/dL to a nadir of 11 g/dL two days after the bleeding; no transfusion was administered. Based on this hemoglobin decrease and in the absence of hemolysis or transfusion, the total blood loss was estimated at ~700-800 mL.

**Figure 4:**
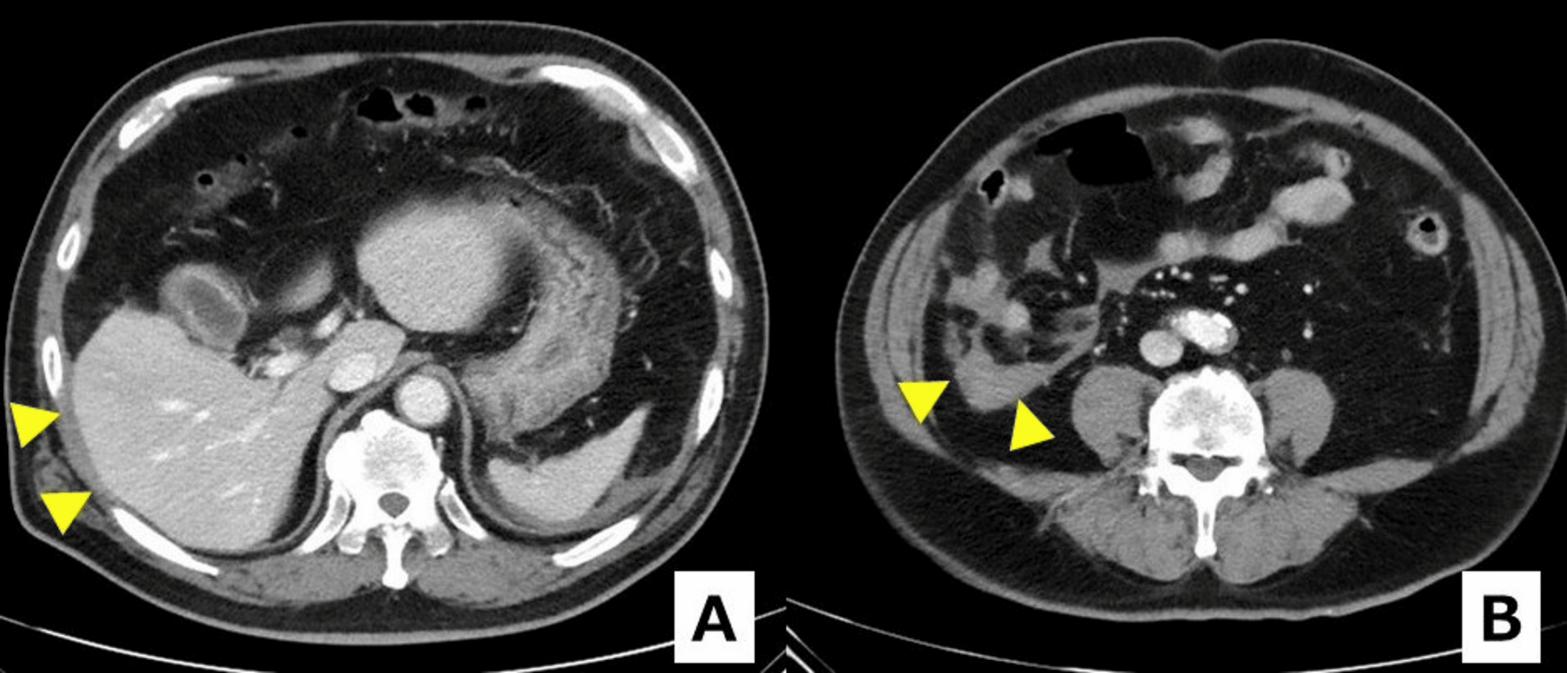
Follow-up contrast-enhanced CT one week after tube dislodgement (A and B) Arrowheads indicate residual bloody ascites that has decreased in volume compared with the prior scan but persists along perihepatic and right paracolic spaces. The attenuation remains above simple fluid (~0-20 HU) and compatible with acute blood (~30-45 HU), again supporting hemoperitoneum, with no mass-like hematoma or organized collection. CT: computed tomography, HU: Hounsfield units

Edoxaban was withheld for seven days after the bleeding event and then restarted while the patient remained clinically stable. A follow-up CT one week later showed decreased but persistent liquid bloody ascites without a mass-like hematoma (Figure 4). The patient was scheduled for interval surgery.

**Figure 5:**
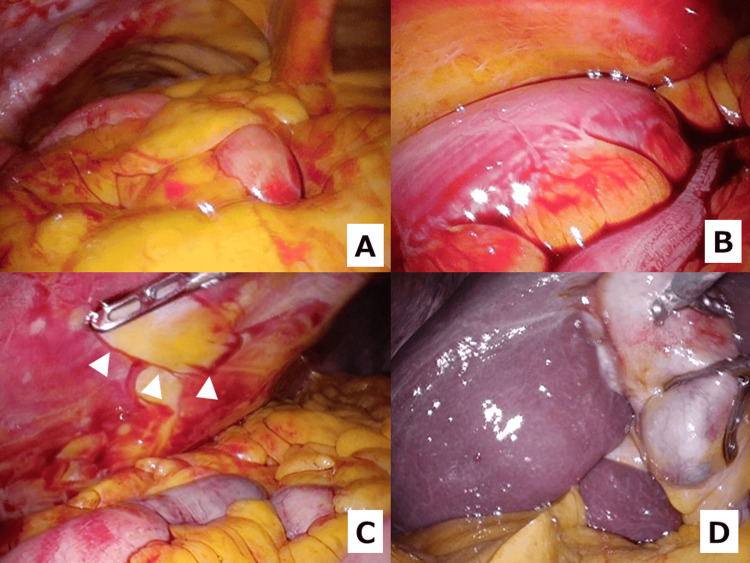
Laparoscopic views during interval cholecystectomy (A) Panoramic survey of the abdominal cavity with free liquid blood visible in dependent areas. (B) Magnified view demonstrating pooling of liquid blood between intestinal loops. (C) Old hemorrhagic residue on the abdominal wall can be wiped away with forceps (arrowheads). (D) View with the gallbladder elevated; no active bleeding is seen from the gallbladder or the hepatic surface.

Approximately one month after the bleeding event, an elective laparoscopic cholecystectomy was performed. At laparoscopy, a panoramic survey revealed free liquid blood in dependent areas (Figure 5A); a magnified view demonstrated pooling between intestinal loops (Figure 5B); old hemorrhagic residue on the abdominal wall could be wiped away with forceps (Figure 5C). With the gallbladder elevated, no active bleeding was seen from the gallbladder or the hepatic surface (Figure 5D).

The hemorrhagic fluid was readily aspirated, and cholecystectomy proceeded without difficulty. These findings required thorough suction-irrigation but did not materially alter the operative plan or technical steps. The operative time was 54 minutes (anesthesia time: 90 minutes); the estimated blood loss was minimal; no transfusion was required. Postoperatively, edoxaban was resumed on postoperative day 1; the clinical course was uneventful, and the patient was discharged on postoperative day 3.

Routine laboratory data obtained during the clinical course are summarized in Table 1. On admission with acute cholecystitis, the patient presented with leukocytosis, elevated C-reactive protein (CRP), and mild hyperbilirubinemia, while coagulation indices (prothrombin time (PT)-international normalized ratio (INR) and activated partial thromboplastin time (APTT)) were only slightly prolonged. After spontaneous dislodgement of the PTGBD tube and the development of hemoperitoneum, serial laboratory tests showed a gradual decrease in hemoglobin and total protein, with a nadir hemoglobin of 11 g/dL on day 2 after bleeding, although no transfusion was required. Platelet counts remained preserved or even elevated, and conventional coagulation indices did not reveal critical derangement at any time point. Renal function and hepatic transaminases remained within or close to reference ranges, except for transient increases in gamma-glutamyl transferase (γ-GT) and alanine aminotransferase (ALT). By one month after the bleeding event, on postoperative day 1 following laparoscopic cholecystectomy, hemoglobin had improved to 14.6 g/dL, and inflammatory markers had normalized. These findings indicate that, despite persistent intraperitoneal liquid blood, systemic coagulation failure or organ dysfunction was not evident throughout the course.

**Table 1:** Laboratory data across the clinical course This table summarizes the laboratory data during the clinical course of the patient. “NP” denotes that the test was not performed at the indicated time point. (H): above reference range, (L): below reference range, NP: not performed, PTGBD: percutaneous transhepatic gallbladder drainage, LC: laparoscopic cholecystectomy, PT: prothrombin time, INR: international normalized ratio, APTT: activated partial thromboplastin time, TP: total protein, T-Bil: total bilirubin, D-Bil: direct bilirubin, AST: aspartate aminotransferase, ALT: alanine aminotransferase, γ-GT: gamma-glutamyl transferase, UN: urea nitrogen, CRE: creatinine, CRP: C-reactive protein

Parameter (unit)	Reference range	Admission, PTGBD	Tube cholangiography	Intraperitoneal bleeding	2 days after bleeding	Edoxaban restarted, 7 days after bleeding	Postoperative day 1 after LC, 1 month after bleeding
WBC (×10²/µL)	35-85	201 (H)	94 (H)	134 (H)	102 (H)	141 (H)	86 (H)
Hb (g/dL)	13-17	16.1	15.7	12.6 (L)	11 (L)	12.8 (L)	14.6
PLT (×10⁴/µL)	13-37	28	28.5	29.4	31.1	50.6 (H)	23.7
PT-%	80-120	73 (L)	NP	NP	NP	72 (L)	NP
PT-INR	0.85-1.15	1.26 (H)	NP	NP	NP	1.25 (H)	NP
APTT (seconds)	25-35	26.9	NP	NP	NP	26.7	NP
TP (g/dL)	6.5-8.2	8	7.1	NP	6.1 (L)	7.2	6.7
T-Bil (mg/dL)	0.2-1.2	1.8 (H)	1.7 (H)	NP	1.1	2.9 (H)	1
D-Bil (mg/dL)	0.0-0.3	0.2	0.1	NP	NP	0.1	0.1
AST (U/L)	13-30	28	26	NP	21	52	44 (H)
ALT (U/L)	7-23	33 (H)	24 (H)	NP	27 (H)	25 (H)	86 (H)
γ-GT (U/L)	10-50	48	84 (H)	NP	123 (H)	53 (H)	233 (H)
UN (mg/dL)	8-20	18.4	12.7	NP	12.3	11	12.9
CRE (mg/dL)	0.60-1.00	0.86	1.01 (H)	NP	1.00	1.02	0.91

## Discussion

This case highlights a distinctive coagulation phenotype under a factor Xa inhibitor: persistent, free-flowing hemoperitoneum at interval laparoscopy roughly one month after the index bleed, despite clinical stability and no ongoing hemorrhage. In the broader context of DOAC therapy, where bleeding patterns shift away from intracranial events toward gastrointestinal and intra-abdominal sites compared with warfarin [1,2], our observations suggest a medication-modified trajectory of intraperitoneal blood. Mechanistically, edoxaban reduces thrombin generation and impairs fibrin polymerization [3]; together with its pharmacokinetic profile (terminal half-life of ~10-14 hours and substantial renal elimination) [13], this can limit secondary coagulation of blood that has already escaped into the peritoneal space, so that collections remain liquid even after hemostasis is secured.

The clinical setting aligns with guideline-based care for high-risk acute cholecystitis: PTGBD is endorsed in the Tokyo Guidelines, although complications include bile leakage, tube dislodgement, and bleeding from the transhepatic tract or gallbladder bed [4,5]. Hemoperitoneum after PTGBD has been described, including severe courses such as autopsy and fatal self-removal reports [6,9,10], and DOAC-associated spontaneous intra-abdominal bleeding has also been reported under factor Xa inhibitors [8,11,12]. What distinguishes the present case is the intraoperative confirmation that hemoperitoneum remained liquid and non-organized for weeks after the presumed bleeding source had ceased.

Our imaging-operative correlation was internally consistent. On CT, unclotted liquid blood typically measures ~30-45 HU, lower than organized clot (~45-70 HU), and ancillary signs (e.g., sentinel clot sign and dependent layering) assist characterization and source localization [14]. Serial scans favored non-organized liquid attenuation without a mass-like hematoma, matching laparoscopy showing large-volume, free-flowing dark blood without active extravasation. Under usual physiology, extravasated blood undergoes clot formation → organization → resorption over days to weeks [3]; deviation from this trajectory supports a DOAC-modified course.

These dynamics carry operative implications. For stable patients, an interval strategy remains reasonable; however, persistent liquid blood at the target field can obscure tissue planes and intended transection lines (e.g., around the gallbladder and within Calot’s triangle), raising the risk of layer misidentification or iatrogenic injury. Pragmatic safeguards include early and repeated suction-irrigation, deliberate patient positioning to drain dependent pools, and deferring dissection until planes are unequivocally visualized; if safe visualization cannot be achieved, conversion or staged management is appropriate [4,5,15]. In unstable bleeding or when hemorrhage persists, peri-procedural guidance supports reversal strategies, with specific reversal (andexanet alfa) for factor Xa inhibitors where indicated and available, alongside definitive hemostasis such as transcatheter arterial embolization [15,16].

Limitations include the absence of anti-Xa levels and peritoneal fibrinolytic markers; thus, causality cannot be proven. Nonetheless, the temporal course under edoxaban [13], the CT attenuation pattern [14], the peri-procedural framework for DOACs [15], and the laparoscopic confirmation together support a DOAC-modified coagulation trajectory in which intraperitoneal blood may remain liquid for weeks after the bleeding event.

## Conclusions

In a patient on edoxaban, hemoperitoneum after PTGBD tube dislodgement persisted in a liquid, non-organized state for about one month and was confirmed at laparoscopy. In anticoagulated patients, surgeons should anticipate persistent liquid collections and operate with the expectation that identifying dissection planes and transection lines may be difficult, preparing for early suction-irrigation as needed.
